# Detection of Neonicotinoid-Contaminated Drinking Water by D‑SERS and SIMCA Class Modeling

**DOI:** 10.1021/acsomega.6c08648

**Published:** 2026-10-13

**Authors:** Gabriela P. Oliveira, Mariana R. Almeida, Gustavo F. S. Andrade

**Affiliations:** † Department of Chemistry, Universidade Federal de Juiz de Fora, José Lourenço Kelmer Street, Juiz de Fora, Minas Gerais 36036-900, Brazil; ‡ Department of Chemistry, Universidade Federal de Belo Horizonte, Antônio Carlos Av., Belo Horizonte, Minas Gerais 31270-901, Brazil

## Abstract

Neonicotinoids are insecticides widely used in agriculture and are highly toxic to invertebrates. There is evidence linking the use of neonicotinoids to declines in bee populations. Due to their high persistence, these compounds can remain in soil and water for long periods and are frequently detected in surface water, groundwater, and even drinking water, thereby posing risks to human health. This study reports the development of a polytetrafluoroethylene (PTFE)-based AgNP substrate for the detection of the neonicotinoids imidacloprid (IMD), thiamethoxam (THX), and their mixture in drinking water using the D-SERS (Dynamic-Surface Enhancement Raman Spectroscopy) methodology, together with the development of a class-modeling method based on Soft Independent Modeling of Class Analogy (SIMCA) that allowed the classification of drinking water samples containing one or more pesticides, achieving an efficiency of 97%. This study presents the first reported application of the SIMCA model for the classification of neonicotinoids.

## Graphical abstract

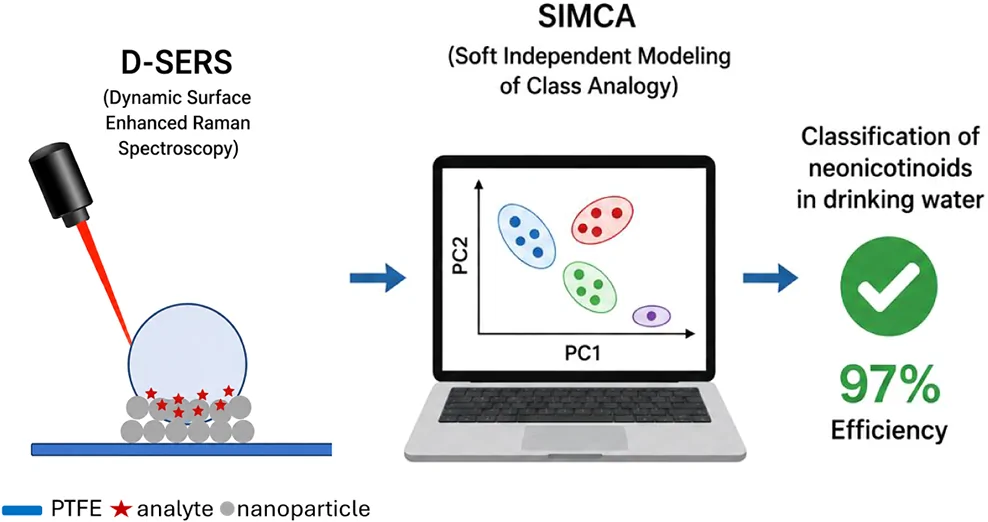

## Introduction

Neonicotinoids are a class of insecticides developed in the 1980s and widely used in agriculture; however, their physicochemical characteristics such as high-water solubility, low soil sorption, and long half-life in soil, contribute to their persistence and transport in the environment, facilitating their transport to water bodies and bioaccumulation in organisms.^1^

In 2019, the joint FAO/WHO (Food and Agriculture Organization of the United Nations/World Health Organization) meeting on pesticide residues established acceptable daily intake levels at 10–200 μg/kg body weight for various neonicotinoids for a lifetime without appreciable health risk.^2^ Numerous studies have demonstrated the contribution of neonicotinoids to pollinator losses, especially in honeybees, but their effects on human health remain inconclusive, although reported incidents suggest adverse effects on the nervous system and brain.^2^ In 2013, the European Food and Safety Authority (EFSA) declared neonicotinoids a high risk to wildlife and honeybees.^2^ The Brazilian Ministry of Health review of drinking water standards established a maximum permissible limit of 72.0 μg L^–1^ (2.5 × 10^–7^ mol L^–1^) for thiamethoxam (THX) in drinking water.^3^ However, no such limit has been established for imidacloprid (IMD) under Brazilian legislation. Therefore, this work adopts the guidelines set by Health Canada, which allows 500 μg L^–1^ (1.9 × 10^–6^ mol L^–1^) for IMD and 5 μg L^–1^ (1.6 × 10^–7^ mol L^–1^) for THX.^4^

Studies have reported the presence of neonicotinoids in surface water samples and drinking water. Imidacloprid, and acetamiprid were detected in all surface water samples throughout the rainy and dry seasons in the Yangtze River Basin, China.^5^ In another study, three neonicotinoids (clothianidin, imidacloprid and, thiamethoxam) were detected at concentrations of up to 57 ng L^–1^ in water collected from the drinking water treatment system at the University of Iowa in the USA.^4^ In this context, it is important to develop rapid and effective methods for detecting traces level of neonicotinoids.

Surface-enhanced Raman spectroscopy (SERS) has been explored as a potential alternative to commonly used methods, such as high-performance liquid chromatography (HPLC) and mass spectrometry, for monitoring neonicotinoid residues, as these established methods are time-consuming and require significant sample preparation.^6^ Furthermore, the SERS technique allows highly sensitive detection also has the advantage of allowing field detection.^7^ Therefore, SERS is a powerful technique for food and environmental analysis.^8–11^ Recent studies utilized SERS for IMD and THX detection.^12–15^ AuNP were used to detect imidacloprid in public water supplies and lake water samples in Madison, USA. The analysis was supported by multivariate analysis, including principal component analysis (PCA),^16^ enabling the detection of IMD down to a concentration of 1.0 × 10^–7^ mol L^–1^.

Several strategies have been developed to improve SERS performance for the analysis of pesticides. Often, plasmonic nanoparticles need to be functionalized to improve their interaction with pesticides,^17^ or it is necessary to use plasmonic nanoparticles that generate more hot spots or to employ aggregation methods.^6^ Obtaining SERS spectra of pesticides remains a significant challenge, even though many have chemical structures that could potentially exhibit high affinity for plasmonic nanoparticles. The intrinsically low Raman scattering cross-section and the small molecular size of most pesticides may be among the factors that hinder their detection.

This study employed polytetrafluoroethylene (PTFE) together with the D-SERS (Dynamic SERS) methodology to facilitate the detection of neonicotinoids. PTFE has been used as a support to construct SERS substrates due to its high reproducibility, high Raman intensification, and surface hydrophobicity, which limits droplet spreading and favors the confinement of nanoparticles and analyte molecules in the region where the droplet is deposited.^18,19^ It also exhibits interesting properties such as chemical stability and mechanical resistance.^20^ These characteristics are particularly advantageous for D-SERS, since the confinement of AgNPs and analyte molecules within a smaller area during droplet evaporation increases their proximity and favors the formation of SERS-active hot spots, improving the detection of analytes at low concentrations. D-SERS is a method developed to collect SERS spectral data during the analyte transition from the wet to the dry state.^21^ As shown in Figure 1, nanoparticles are deposited on a hydrophobic support, and a droplet containing the analyte is placed on the substrate (Figure 1(a)). During solvent evaporation, the nanoparticles and analytes reorganize (Figure 1(b)) and adopt dynamic orientations, favoring higher SERS intensities while minimizing thermal decomposition of the analyte. In the dry state (Figure 1(c)), when the solvent has completely evaporated, the hot spots are fixed in random orientations, which is an unfavorable configuration to the SERS effect,^22^ and the laser incidence would also increase thermal effects. In the literature, the D-SERS methodology has been widely used to detect pesticides at low concentrations using filter paper substrates^22^ and silicon substrates.^23,24^ However, PTFE offers additional advantages as a support for D-SERS due to its hydrophobic surface, which restricts droplet spreading and confines the AgNPs and analyte molecules to a smaller area during evaporation. This confinement favors the internanoparticle and adsorbate-nanoparticle interactions and the formation of SERS-active hot spots, making PTFE particularly advantageous for D-SERS applications compared with traditional supports.

**Figure 1:**
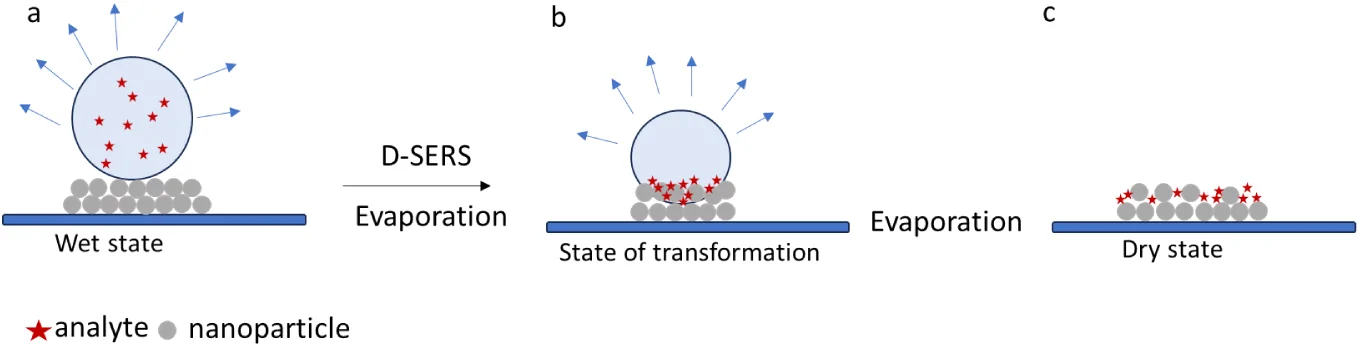
Schematic illustration of the evaporation process during D-SERS (a) wet state (b) transformation state (c) dry state.

The detection of organic molecules in real samples by SERS may be challenging, and approaches involving chemometrics have been extensively studied in recent years.^25^ Sample preparation and data analysis are two key aspects to consider when evaluating the feasibility of quantification using SERS.^26^ However, several challenges still need to be addressed for reliable quantitative analysis. In this context, qualitative analyses may prove more efficient and robust than quantitative approaches. Multivariate classification methods, such as class modeling, can be employed to determine whether a sample belongs to a target class, thereby enabling the detection of the presence or absence of a specific contaminant.^27^ Teixeira and Poppi prepared filter paper-based substrates using gold nanoparticles for the detection of the pesticide thiabendazole in water extracts of mango peel combined SERS with Data Driven Soft Independent Modeling of Class Analogies (DD-SIMCA). The use of the SERS technique together with DD-SIMCA provided a potential way to classify samples with significant levels equivalent to those stipulated by the Brazilian Health Regulatory Agency, showed good selectivity (94%) and sensitivity (92%).^28^

In this work, SIMCA (Soft Independent Modeling of Class Analogy)^29^ class modeling method was employed to classify drinking water samples as either pesticide- spiked or nonspiked based on SERS spectra. Flexible PTFE–AgNP substrates and D-SERS methodology were investigated to provide a stable and reliable platform for spectral acquisition, minimizing sample degradation effects and enhancing the SERS response of the target pesticides. The model was built using samples containing imidacloprid, thiamethoxam, and a mixture of both pesticides. To the best of our knowledge, this is the first study to report the combined use of SERS and SIMCA for neonicotinoid classification, highlighting the novelty of this approach and its potential as a rapid analytical screening strategy.

## Experimental Section

### Chemicals

The chemicals silver nitrate (Sigma-Aldrich, 99.0%), sodium borohydride (Sigma-Aldrich, 98.0%), hydrochloric acid (Synth, 37%), polytetrafluoroethylene (PTFE) membrane (Allcrom), imidacloprid (Sigma-Aldrich), thiamethoxam (Sigma-Aldrich) were used without any further purification. Aqueous solutions for substrate development and standard sample testing were prepared using deionized water from Millipore Synergy-UV system with resistivity 18.2 MΩ cm. Drinking water was obtained from taps connected to the local water supply system from the city of Juiz de Fora, Brazil; details are given below.

### AgNP Synthesis

The AgNP synthesis followed the procedure reported by Emonds-Alt et al.,^30^ adapted from the procedure proposed by Creighton.^31^ Initially, the reducing agent NaBH_4_ (150 mL, 2.0 × 10^–3^ mol L^–1^) was added to an ice bath under vigorous stirring. AgNO_3_ (50 mL, 2.5 × 10^–3^ mol L^–1^) was added dropwise and stirring was maintained for 30 min.^32^ The resulting suspension presented a brown coloration. After cooling down to room temperature, it was stored at 4 °C for later use.

### Substrate Preparation and Deposition of Pesticides for the SERS Experiments

To prepare the SERS substrates on PTFE supports, 2.0 mL of the AgNP suspension was centrifuged at 6100 rpm for 30 min. After 1.8 mL supernatant removal, the AgNP concentrated was reserved, and subsequently deposited onto a PTFE membrane using drop casting (10 μL); after deposition, the AgNP-PTFE membrane was dried in an oven at 50 °C. Five deposition steps of the colloidal suspension were made for each PTFE membrane.

### Characterization of Substrates and SERS Spectroscopic Measurements

The AgNP were characterized by UV–vis spectroscopy using the Ocean Optics 2000 + XR1 + ES spectrometer in the range 200–1800 nm (Figure S1). Scanning electron microscopy (SEM) micrographs were obtained for the AgNP-PTFE substrates using a double beam electronic, and ionic FEI Quanta 3D FEG (Figure S2). Experimental and analysis were performed in the Center of Microscopy of the Universidade Federal de Minas Gerais, Belo Horizonte, Brazil (http://www.microscopia.ufmg.br)

The SERS analyses were performed using a Bruker SENTERRA spectrometer with the laser line at 633 nm of a HeNe laser, using a 50× magnification objective (NA = 0.50), 0.2 mW laser power and 60 s of signal accumulation. A low laser power was employed to minimize laser-induced degradation of the samples due to thermal effects observed at higher laser powers. Consequently, a longer integration time was used to obtain spectra with a high signal-to-noise ratio.

### Temporal Stability of the SERS Performance of the AgNP-PTFE Substrates

Ag-PTFE substrates were prepared in triplicate over 45 days, with a 15-day interval, and the gentian violet molecule (1.0 × 10^–5^ mol L^–1^) was used to evaluate the temporal stability of the substrates. The measurements were taken after 45 days, maintaining the same spectral parameters. The spectra were baseline-corrected followed by Standard Normal Variate (SNV) preprocessing. Subsequently, the band at 915 cm^–1^ was integrated, and the resulting integrated area values were used to construct the bar graph.

### SERS Analysis in Drinking Water

Two collection points were selected in the city of Juiz de Fora, Minas Gerais, Brazil. Water samples were collected on different days at the two collection points, directly from taps connected to the public water supply points. The water samples were centrifuged for 10 min at 14.000 rpm, half of the supernatant was removed, and the supernatant was then filtered using a 0.22 μm PES membrane filter. For the SERS studies, the sample was spiked with pesticides, and dilutions were performed. A stock solution of each pesticide at 1.0 × 10^–3^ mol L^–1^ was initially prepared and subsequently diluted to obtain final concentrations ranging from 1.0 × 10^–4^ mol L^–1^ to 1.0 × 10^–7^ mol L^–1^.

### Data Processing

An exploration model (PCA) and a classification model, SIMCA, were constructed using Matlab, version 7.10 (The MathWorks, Natick, MA, EUA) and the PLS Toolbox version 5.2.2 (Eigenvector Research, Manson, WA, USA). The spectral data underwent different types of preprocessing to obtain the best result for the model. The training set was used to select the most appropriate preprocessing method, whereas the test set was used exclusively to evaluate the performance of the final model. The preprocessing methods tests were: 1) WLS (Weighted Least Squares) followed by SNV (Standard Normal Variate), or WLS followed by MSC (Multiplicative Scatter Correction), then SNV; 2) first derivative; 3) data were mean-centered (MC).^33^ The model was evaluated using the values of sensitivity, specificity and efficiency. Their mathematical definitions are given eqs 1, 2, and 3. For the SIMCA model, the 51 samples containing pesticides were divided into a training set, a test set using Kennard–Stone algorithm,^34^ which ensures the representativeness of the samples. The number of independent substrates analyzed for each pesticide concentration is presented in Table S1 (Supporting Information). The target class being modeled consists of samples containing pesticides. The algorithm allowed 34 samples to be selected for the training set and 17 for the test set, all containing pesticides. After modeling, samples of water without pesticides were added to the test set.

1 $$\mathrm{Sensitivity}=\frac{TP}{TP+FN}\times 100$$

2 $$\mathrm{Specificity}=\frac{TN}{TN+FP}\times 100$$

3 $$\mathrm{Efficiency}=\frac{TP+TN}{TP+TN+FP+FN}\times 100$$

## Results and Discussion

### Temporal Stability of the SERS Performance of the AgNP-PTFE Substrates

For the substrates stability study, monitoring was carried out over a 45-day period using the violet gentian (VG).^35^ The band at 915 cm^–1^ was chosen to perform the mean and standard deviation analyses, based on the maximum intensity of the band. The SERS spectra of VG were obtained on the same day for the batch of substrates, under the same spectral conditions for all samples. Figure 2(a) shows the SERS spectra of the VG (1.0 × 10^–5^ mol L^–1^) obtained for a selected replica of each substrate. One may observe that the substrate produced with the longest interval before the experiments presents a lower SERS signal than the substrate produced 1 day before the SERS measurements. On the other hand, it is interesting to note that the SERS spectra of VG present similar intensity for the substrates prepared between 15 days and 45-days, with some intensity fluctuations. Figure 2(b) shows the bar graph with the relative standard deviation calculated for each set of substrates evaluated. We can observe that the substrates stored for 45 days showed a lower average intensity, while the average intensities for the substrates stored for 30 days, 15 days, and 1 day were similar

**Figure 2:**
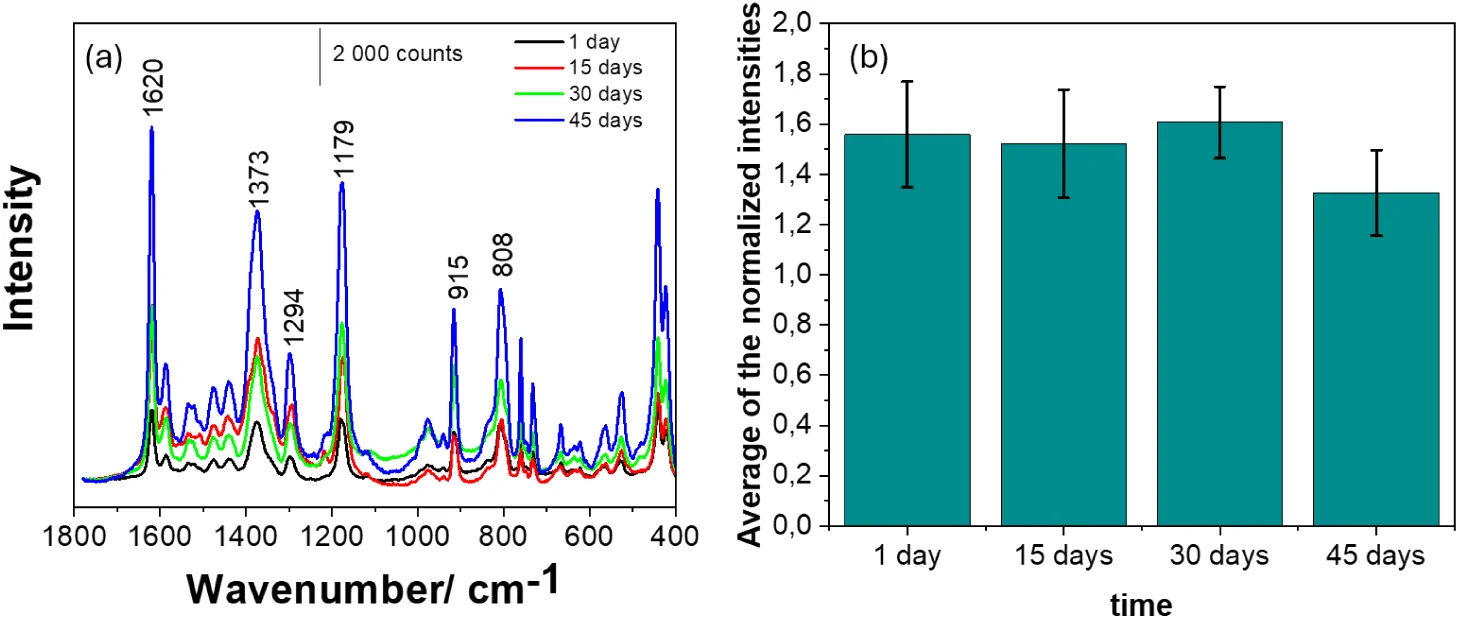
(a) SERS spectra of violet gentian (1.0 × 10^–5^ mol L^–1^) on Ag-PTFE substrate with different storage times 1 day to 45 days (b) Average integrated area of the band at 915 cm^–1^ of violet gentian with relative standard deviation with different storage times.

Finally, we can conclude that the substrates exhibit good stability and can be produced well in advance of SERS experiments and stored, provided they are kept in a dry environment at near ambient temperature and low light and can be used for subsequent analysis.

### SERS of IMD and THX

Figure 3 presents the SERS spectra of IMD on the AgNP-PTFE substrate at different concentrations. In these spectra, the IMD characteristic bands were observed at 1455, 1289, 1109, 1033, 908, and 824 cm^–1^. The band at 1455 cm^–1^ is assigned to δ­(HCH). The band at 1109 cm^–1^ is assigned to δ­(HCC) and ν­(NC), the band at 1033 cm^–1^ is assigned to ν­(NC), ν­(ClC) and δ­(HCC) the band at 908 cm^–1^ is assigned to ν­(NN), ν­(ON) of the nitro group.^36^ The Raman spectrum of solid IMD is presented in Figure S5 (see the Supporting Information file).

**Figure 3:**
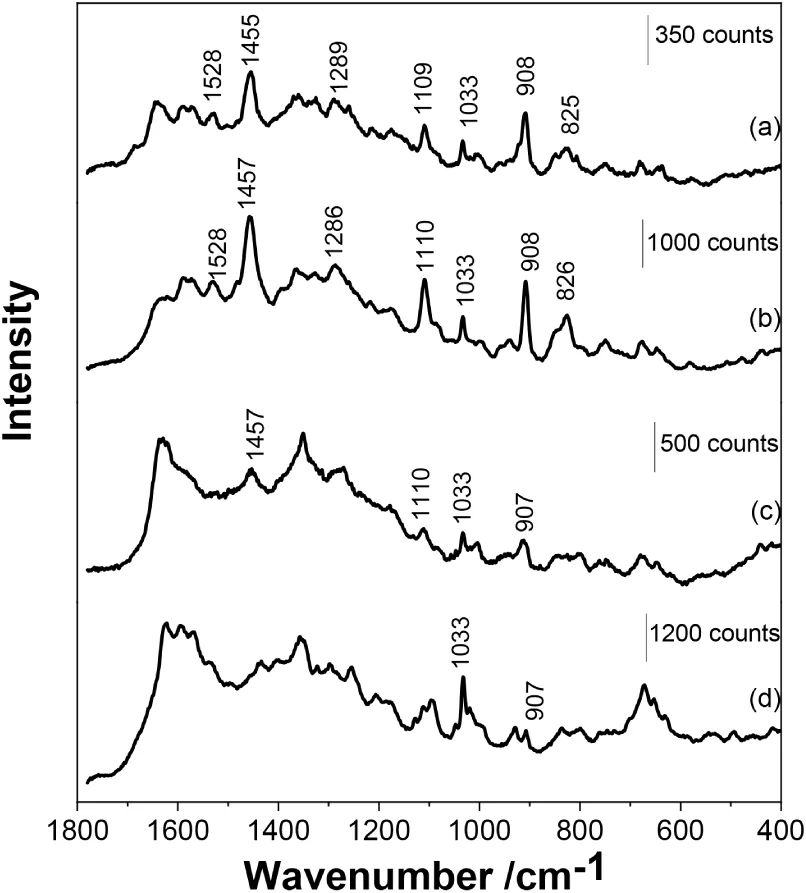
SERS spectra of IMD on Ag-PTFE substrate with different concentrations (a) 1.0 × 10^–3^ mol L^–1^ (b) 1.0 × 10^–4^ mol L^–1^ (c) 1.0 × 10^–5^ mol L^–1^ (d) 1.0 × 10^–6^ mol L^–1^.

It is observed that the intensity of the IMD bands decreases with concentration, but they are observable up 1.0 × 10^–6^ mol L^–1^, below this concentration, it is not possible to detect the characteristic IMD bands, the bands exhibit low intensity, making them difficult to identify without the use of chemometric tools. However, one may notice an increased SERS intensity in the spectrum in Figure 3b compared to the spectrum obtained at a higher concentration in Figure 3a. This variation may be attributed to the dynamic nature of the D-SERS procedure, which may result in intensity fluctuations associated with the density and nature of SERS hot-spots probed during the spectrum acquisition. Furthermore, characteristic bands of amorphous carbon are observed, characteristic of the degradation of organic compounds through the laser used in the measurements. The characteristic bands of PTFE at 732, 1300, and 1380 cm^–1^ were not observed, so the support did not interfere with the detection of the pesticide. The Raman spectra of PTFE and PTFE-AgNP are present in the Supporting Information (Figure S3 and Figure S4).

Figure 4 presents the SERS spectra of THX at concentrations ranging from 1.0 × 10^–3^ mol L^–1^ to 1.0 × 10^–6^ mol L^–1^. The most intense bands are observed at 1584, 1469, 1244, 1042, 998, 758, 637 cm^–1^ are characteristic of THX. It is noticed that the spectra present a high signal/noise ratio, higher than what was observed for IMD. The band at 1584 cm^–1^ is assigned to ν­(NC), the band at 1469 cm^–1^ is assigned to ν­(NC) and τ­(HCCS), the band at 1042 cm^–1^ is assigned to ν­(NN), ν­(NC), the band at 998 cm^–1^ is assigned to τ­(HCCS), ν­(NC), δ­(HCC) and the band at 758 cm^–1^ is assigned to ν­(SC), δ­(NCC).^36^ The Raman spectrum of solid THX is presented in Figure S5 (Supporting Information).

**Figure 4:**
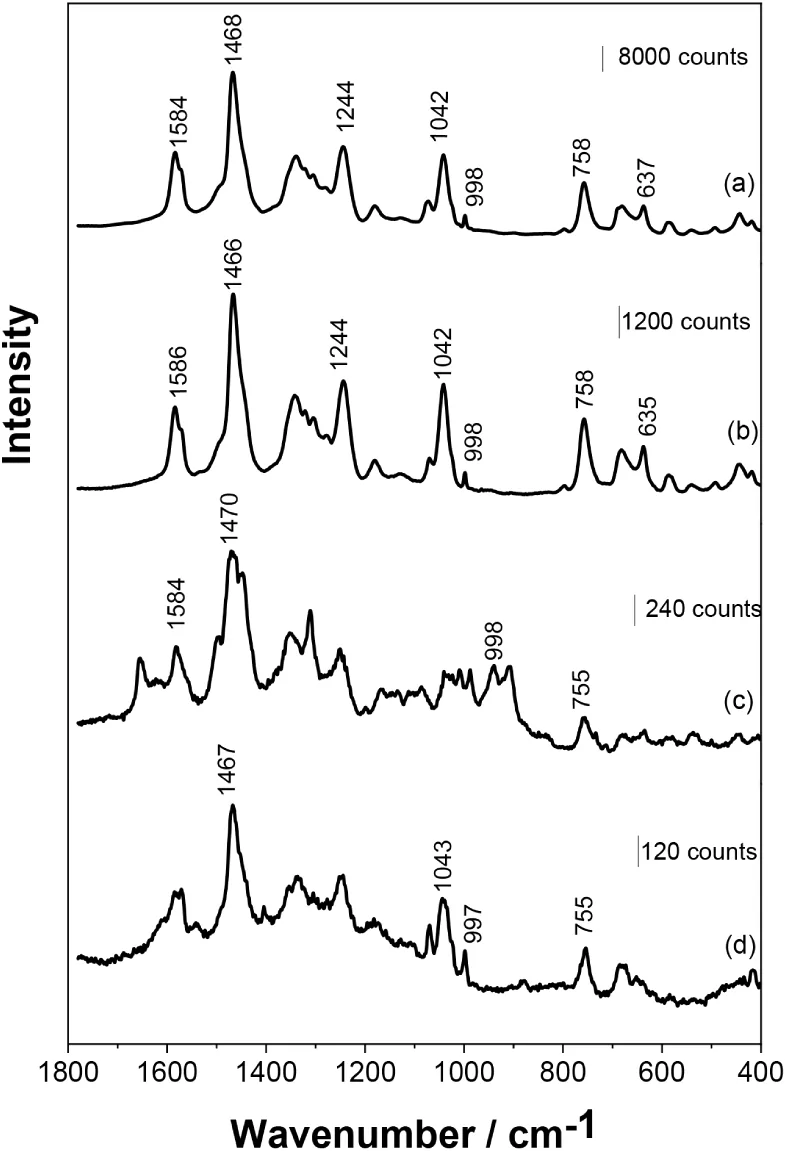
SERS spectra of THX on Ag-PTFE substrate with different concentrations (a) 1.0 × 10^–3^ mol L^–1^ (b) 1.0 × 10^–4^ mol L^–1^ (c) 1.0 × 10^–5^ mol L^–1^ (d) 1.0 × 10^–6^ mol L^–1^.

In Figure 4, it is noted that the intensity of the SERS bands decreases with concentration, in the same way as for the IMD, the formation of a background in the spectrum is observed with lower concentrations. It was possible to detect the characteristics bands of THX up to concentrations 1.0 × 10^–6^ mol L^–1^; at lower concentrations, the bands exhibit low intensity, making them difficult to identify without the use of chemometric tools.

In addition, the SERS spectra of IMD and THX mixtures prepared in deionized water at a concentration of 1.0 × 10^–4^ mol L^–1^, with different IMD ratios (1:1, 1:3, and 3:1), are presented in Figure S6 of the Supporting Information.

### One-Class Model to Detect IMD and THX in Drinking Water

The SERS spectra of IMD, THX, and mixtures (1:1 THX:IMD, 1:3 THX:IMD, 3:1 THX:IMD) of both substances in drinking water were obtained in concentrations ranging from 1.0 × 10^–7^ to 1.0 × 10^–4^ mol L^–1^ on the AgNP-PTFE substrates. The data treatment of the SERS spectra consisted, initially, of an exploratory data analysis that was performed using PCA to visualize the data profile and groupings. Figure 5(A) presents the scores plot obtained for the PCA model with two principal components that explain 68.32% of the total variance of the data. Two anomalous samples were removed, which presented high residual Q values and high T^2^ values (Figure S7, Supporting Information). In the graph presented in Figure 5(A) it is possible to observe a tendency for the samples to group. On the negative side of PC2, most of the samples containing imidacloprid and thiamethoxam are distributed, whereas on the positive side, some samples of the pesticide mixture and most pesticide-free drinking water samples are located. Along PC1, most samples containing pesticides are positioned on the negative side, while a smaller number are distributed on the positive side. The negative side of PC1, is predominantly associated with samples containing pesticides at higher concentrations, although some lower-concentration samples are also present. In contrast, the positive side of PC1 contains a greater proportion of samples with lower pesticide concentrations. Furthermore, the fortified samples, which are closer to the blank, correspond to the lowest concentration evaluated (1.0 × 10^–7^ mol L^–1^.Therefore, a clear separation can be observed between pesticide-containing and pesticide-free drinking water samples, as well as among pesticide-containing samples according to concentration level. The PCA results suggest that the SIMCA model is likely to achieve satisfactory classification performance.

**Figure 5:**
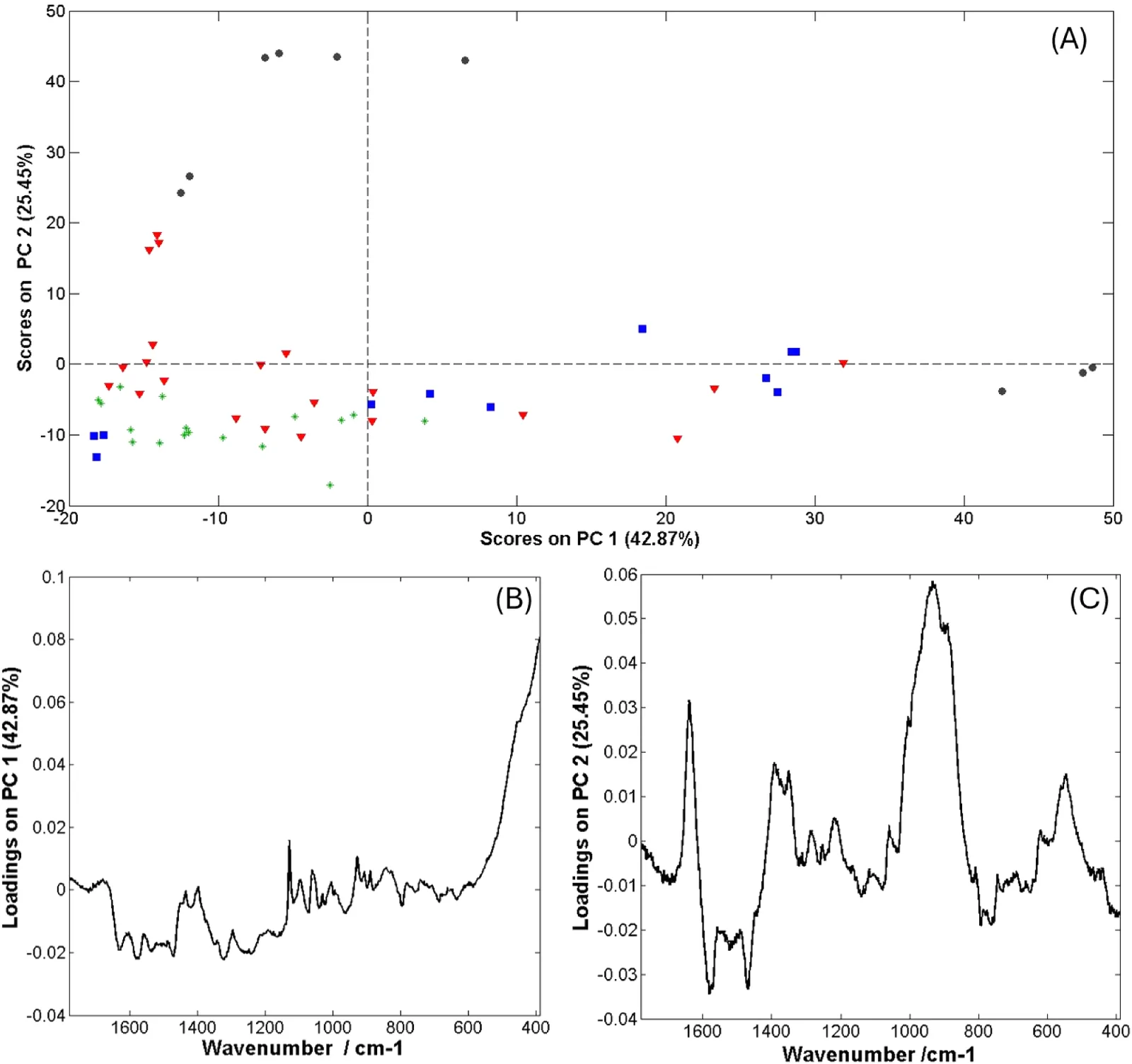
(A) Principal component analysis for 2 PCS of SERS data for pesticides in drinking water and drinking water. IMD (▼red), THX (*Green), mixture (■blue) e blank (•). (B) Loadings on PC1 for the constructed PCA model (C) Loadings on PC2 for the constructed PCA model.

The graph of loadings vs wavenumber (Figure 5(B) and Figure 5(C)) provides information about the variables (bands) that were important and influenced the grouping tendency of the samples. We can observe the main pesticide bands in the PC1 loadings, while the PC2 loading shows bands related to the unfortified sample.

An important step in building the SIMCA model was data preprocessing. Different types of preprocessing were tested using the training set to select the most appropriate preprocessing method, whereas the test set was used exclusively to evaluate the performance of the final model. The evaluation was the performance metrics sensitivity, specificity, and efficiency, which were calculated and are presented in Table 1. As we can observe in the table, the best models were constructed using the following preprocessing methods: WLS + SNV + MC and WLS + MSC + MC. From these models, it was possible to obtain 97% efficiency for the test sets of both models. The WLS + SNV + MC preprocessing was selected for the construction of the model for the analysis of the samples, based on the values obtained for the figures of merit present in Table 1, furthermore, the use of SNV preprocessing can be justified because it is less susceptible to the occurrence of outlier samples.

**Table 1:** Performance Metrics for the Preprocessing Methods Used for the Training and Test Sets

		Training	Test		
Pre processing^a^	PCs	Sensitivity	Sensitivity	Specificity	Efficiency
WLS + SNV + MC	3	1.00	0.94	1.00	0.97
WLS + MSC + MC	3	0.94	0.94	1.00	0.97
WLS + normalize + MC	3	1.00	1.00	0.93	0.97

a WLS = weighted least-squares, SNV = standard normal variation, MSC = multiplicative scatter correction e MC = mean center.

Fifty-one samples containing both types of pesticides and the mixture, as well as 12 samples of pesticide-free water, were selected. The set of samples containing pesticides was divided into training and test sets. The pesticides-free water samples were concatenated to the test set. Figure 6 shows the classification of imidacloprid, thiamethoxam, and the equimolar mixture of both analytes with concentrations ranging from 1.0 × 10^–4^ mol L^–1^ to 1.0 × 10^–7^ mol L^–1^ in drinking water matrix and pure drinking water samples.

**Figure 6:**
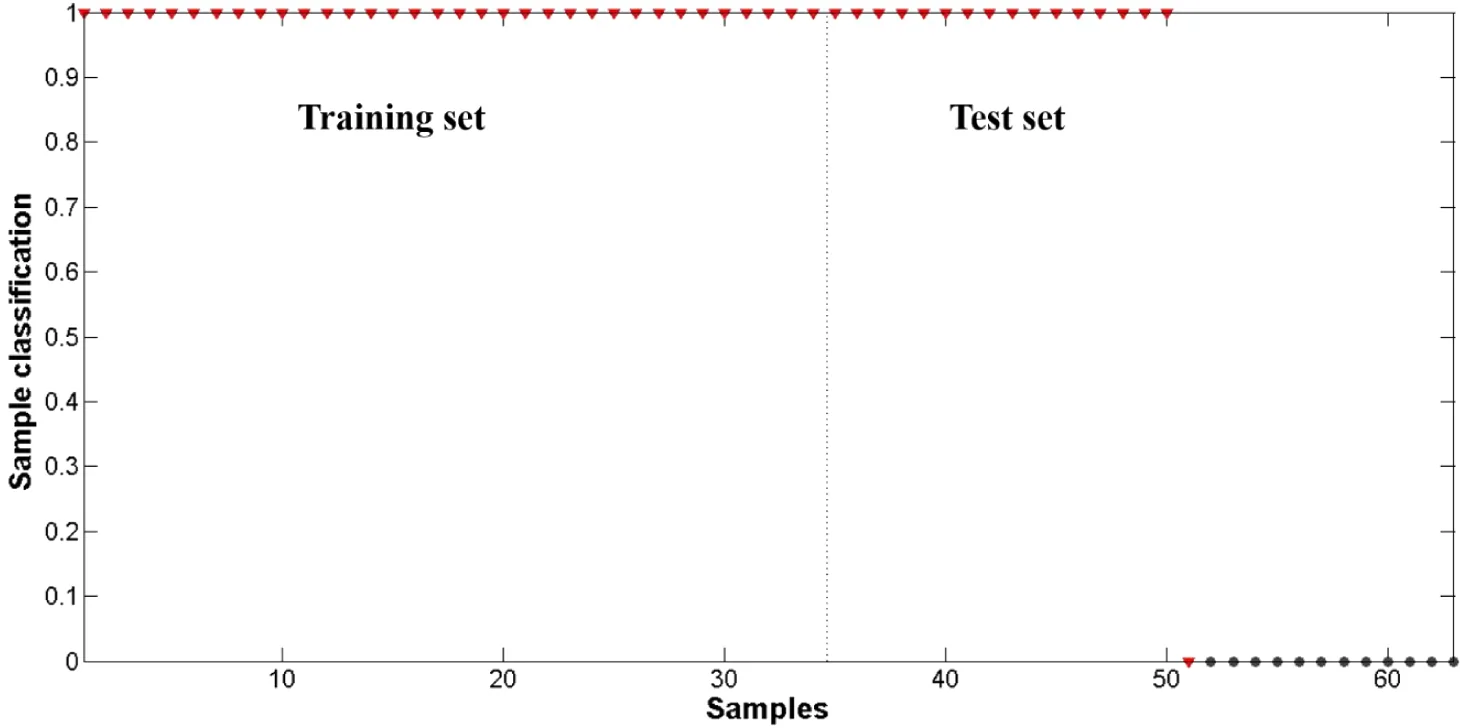
SIMCA model for SERS spectra of pesticides imidacloprid, thiamethoxam and mixture in drinking water, where (▼red) represents samples with pesticides and (•) represents samples without pesticides.

Figure 6 shows that for the training set, all samples containing pesticides were correctly classified. However, in the test set, which contains samples of water with and without pesticides, one sample belonging to the target class was misclassified (false negative) and no samples not belonging to the target class were misclassified (false positive). The misclassified sample was a 1.0 × 10^–4^ mol L^–1^ IMD sample, which may be associated with experimental variability during sample preparation or spectral acquisition. The model achieved 100% specificity, reflecting its capacity to correctly reject every sample belonging to the nonpesticides class. However, this specificity should not be interpreted as evidence of chemical selectivity against other pesticides or interfering species potentially present in environmental water. The good performance of these parameters resulted in a model with 97% efficiency.

The pesticides were detected in concentrations below the maximum limits established by the Brazilian regulations (2.5 × 10^–7^ mol L^–1^ for thiamethoxam). This finding highlights the potential of combining SERS and SIMCA as a rapid and sensitive screening strategy for monitoring neonicotinoid contamination in drinking water. The ability to classify between pesticide-containing and pesticide-free samples at such low concentration levels demonstrates the suitability of this approach for preliminary screening applications, allowing suspect samples to be rapidly identified for subsequent confirmatory analysis.

To contextualize the performance of the proposed method, Table 2 compares the present D-SERS/SIMCA approach with previously reported SERS-based methods for the detection of IMD and THX, considering the SERS substrate, sample matrix, analytical approach, and detection limit or lowest detected concentration. As shown in Table 2, some previously reported methods achieved lower detection limits or lowest detected concentrations than those obtained in the present study. These differences can be attributed to several factors, including the use of highly engineered or functionalized SERS substrates, analyte-specific recognition strategies, sample extraction procedures, and quantitative regression models such as PLS and PLSR. In contrast, the present work was primarily designed as a qualitative screening approach based on D-SERS combined with SIMCA class modeling, aiming to classify water samples according to the presence or absence of neonicotinoids rather than to achieve the lowest possible detection limit. The main advantage of the proposed approach is the combination of D-SERS and SIMCA class modeling as a rapid screening tool for distinguishing water samples containing neonicotinoids from pesticide-free samples, without relying on quantitative calibration models.

**Table 2:** Comparison of Recently Reported SERS-Based Approaches for Neonicotinoid Detection

Neonicotinoid	SERS substrate	Matrix	Chemometric method	Lowest detected concentration	Refs
IMD	Ag-NF	Green tea	PLS, Si-PLS, Bi-PLS, GA-PLS	4.55 × 10^–5^ μg mL^–1^	^37^
IMD and THX	AgNs	Standard solution	PLS regression	IMD: 9.4 × 10^–5^ mg mL^–1^	^38^
				THX: 4.83 × 10^–6^ mg mL^–1^	
IMD	AgNSs	Standard solution/honey, after QuEChERS	PLSR	0.02 μmol L^–1^	^14^
IMD	AuNP	Water	PCA	0.001 μmol L^–1^	^16^
THX	Au@pBDT-TA	Tea	-	Black tea: 0.06 ng g^–1^	^39^
				Green tea: 0.10 ng g^–1^	
IMD and THX	PTFE-AgNP	Water	SIMCA	1.0 × 10^–7^ mol L^–1^	This work

To the best of our knowledge, no previous studies have employed class-modeling approaches, such as SIMCA, in combination with SERS for the classification of these pesticides. Therefore, the proposed methodology represents an innovative contribution to the development of rapid screening tools for water quality monitoring.

## Conclusion

The methodologies presented in this study offer powerful and reliable approach for the identification and classification of neonicotinoid pesticides in drinking water, with potential for application in environmental monitoring and water quality assessment. The PTFE–AgNP flexible substrates showed good stability during the period analyzed, with low variability in analytical response, indicating that they can be prepared in advance and stored for future use without compromising performance. The Dynamic SERS methodology was essential for collecting spectra, as it reduces the effect of sample degradation and intensifies the SERS signal of the pesticides.

It is important to emphasize that the results obtained in this work show that the proposed model can identify thiamethoxam at concentrations below the regulatory limits established by the Brazilian and Canadian agency, and imidacloprid at concentration below the limits established by Canadian agency, as determined using the SIMCA class modeling method with 97% efficiency, demonstrating the capability of the methodology to classify between pesticide-containing and pesticide-free drinking water samples, even at very low concentration levels. These results highlight the potential of combining SERS spectroscopy with SIMCA as a rapid, sensitive, and cost-effective screening strategy for the preliminary assessment of neonicotinoid contamination in water samples.

A particularly novel aspect of this work is the application of class-modeling chemometric strategies (SIMCA) in combination with SERS spectroscopy to identify the neonicotinoid pesticides thiamethoxam and imidacloprid in drinking water samples. To the best of our knowledge, there are no previous reports in the literature of using class modeling with these pesticides in water samples, representing an important contribution to the development of rapid screening methodologies for environmental monitoring.

## Supplementary Material



## Notes

UV–Vis extinction spectrum of the AgNP aqueous colloidal suspension; SEM micrographs of the PTFE membrane and PTFE–AgNP substrate; Raman spectra of the PTFE membrane and PTFE–AgNP substrate; Raman spectra of solid imidacloprid and thiamethoxam; D-SERS spectra of IMD and THX mixtures at different proportions; Q residuals versus Hotelling’s T^2^ plots for PCA outlier evaluation; number of independent substrates analyzed at each pesticide concentration (PDF)
