# Supplementary material for: Detection of Neonicotinoid-Contaminated Drinking Water by D‑SERS and SIMCA Class Modeling
Source: ACS Omega. 2026 Oct 13;11(42):65627–35. doi: 10.1021/acsomega.6c08648 (PMC13717789; doi:10.1021/acsomega.6c08648)
Supplement: Supplementary file 1 [file ao6c08648_si_001.pdf]

Supporting Information for

Detection of Neonicotinoid-Contaminated Drinking Water by D-SERS and SIMCA Class Modeling

*Gabriela P. Oliveira [a], Mariana R. Almeida [b] *, Gustavo F. S. Andrade [a] *.*

[a] Laboratório de Nanoestrutura Plasmônicas, Núcleo de Espectroscopia e Estrutura Molecular, Departamento de Química, Universidade Federal de Juiz de Fora, R. José Lourenço Kelmer, s/n, 36036-900, Juiz de Fora, Brazil.

[b] Departamento de Química, Universidade Federal de Minas Gerais, Belo Horizonte, Brazil.

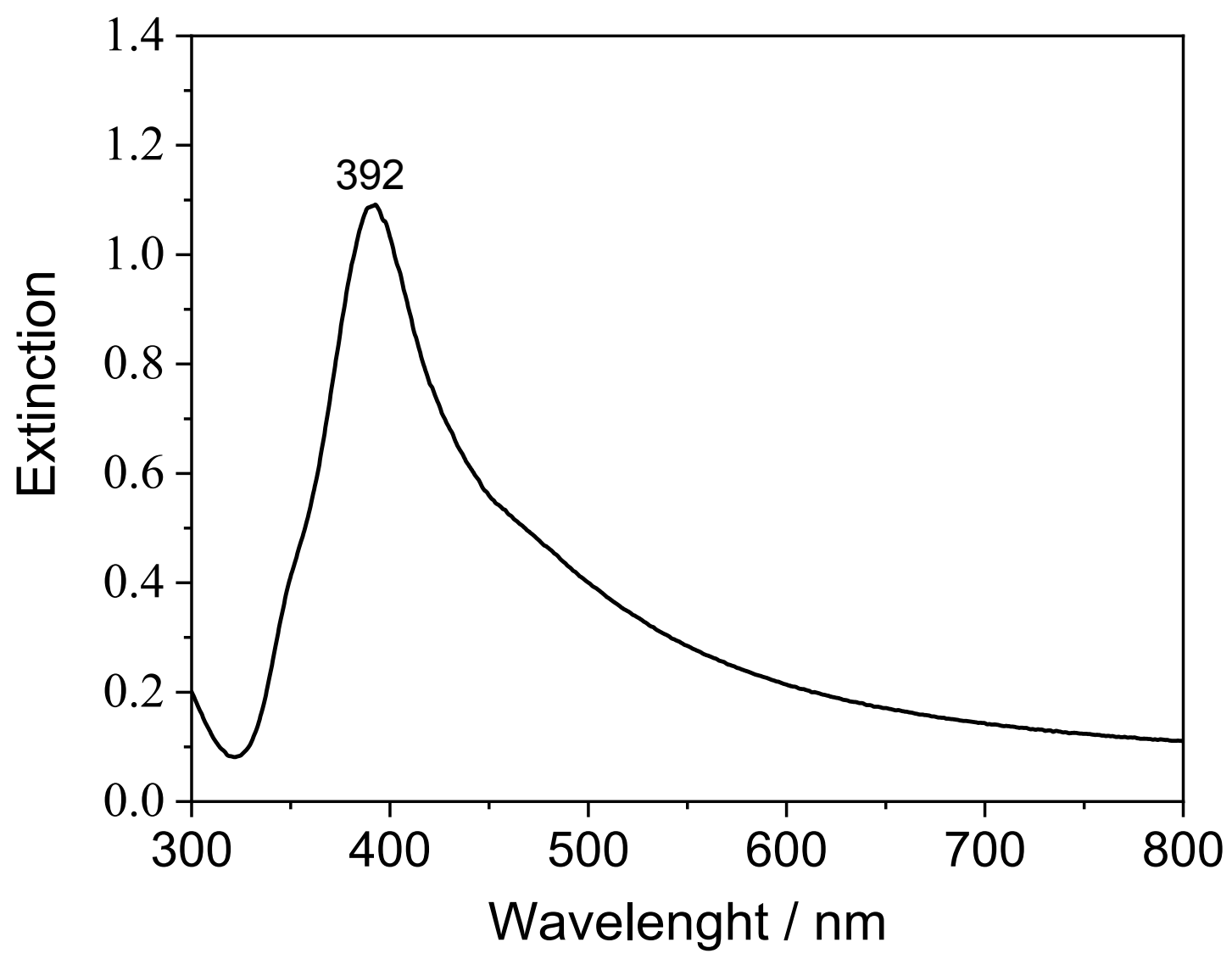

Figure S1: UV-VIS extinction spectrum of AgNP aqueous colloidal suspension.

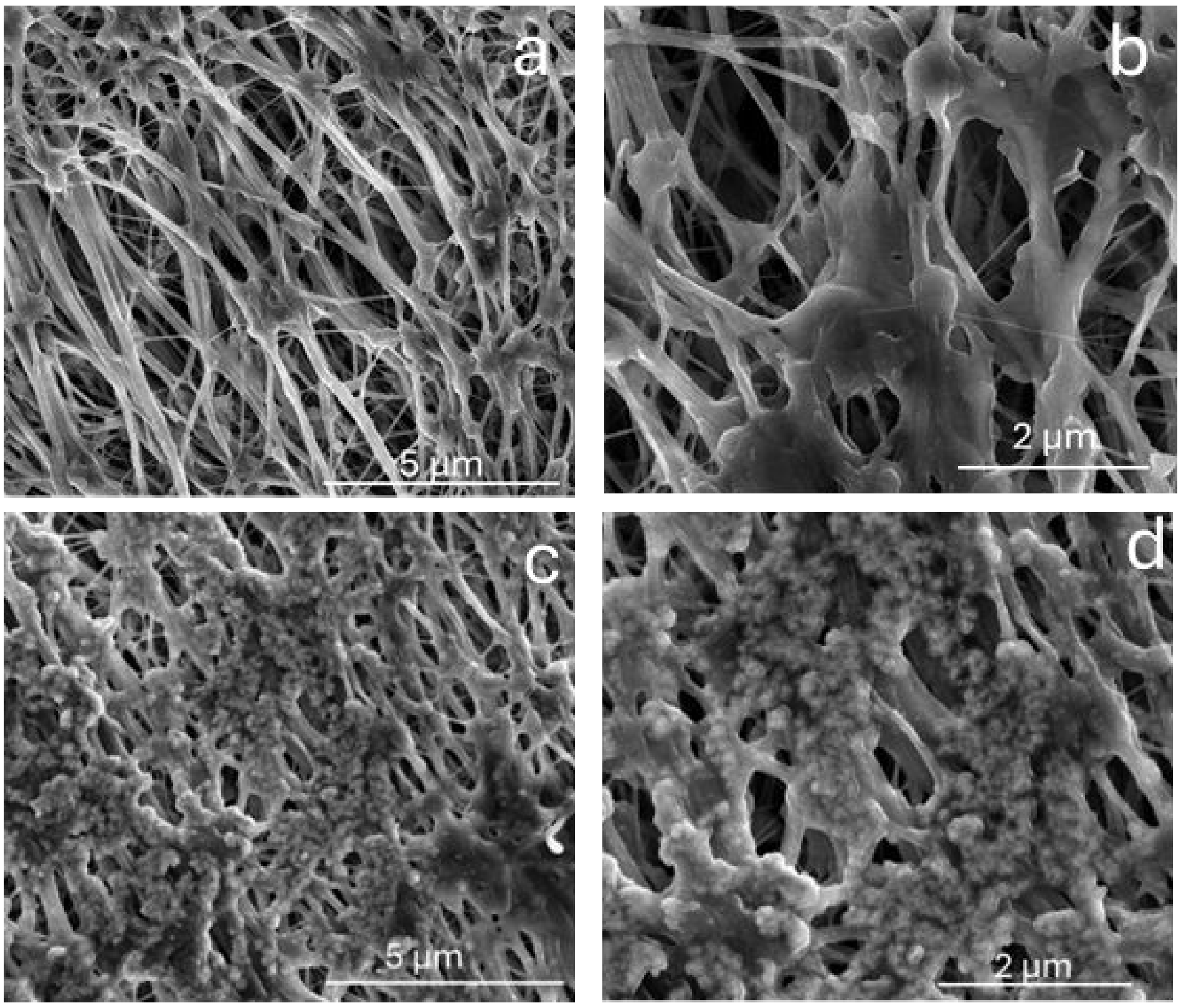

Figure S2: SEM micrographs of (a) and (b) PTFE membrane (c) and (d) PTFE membrane with five AgNP depositions.

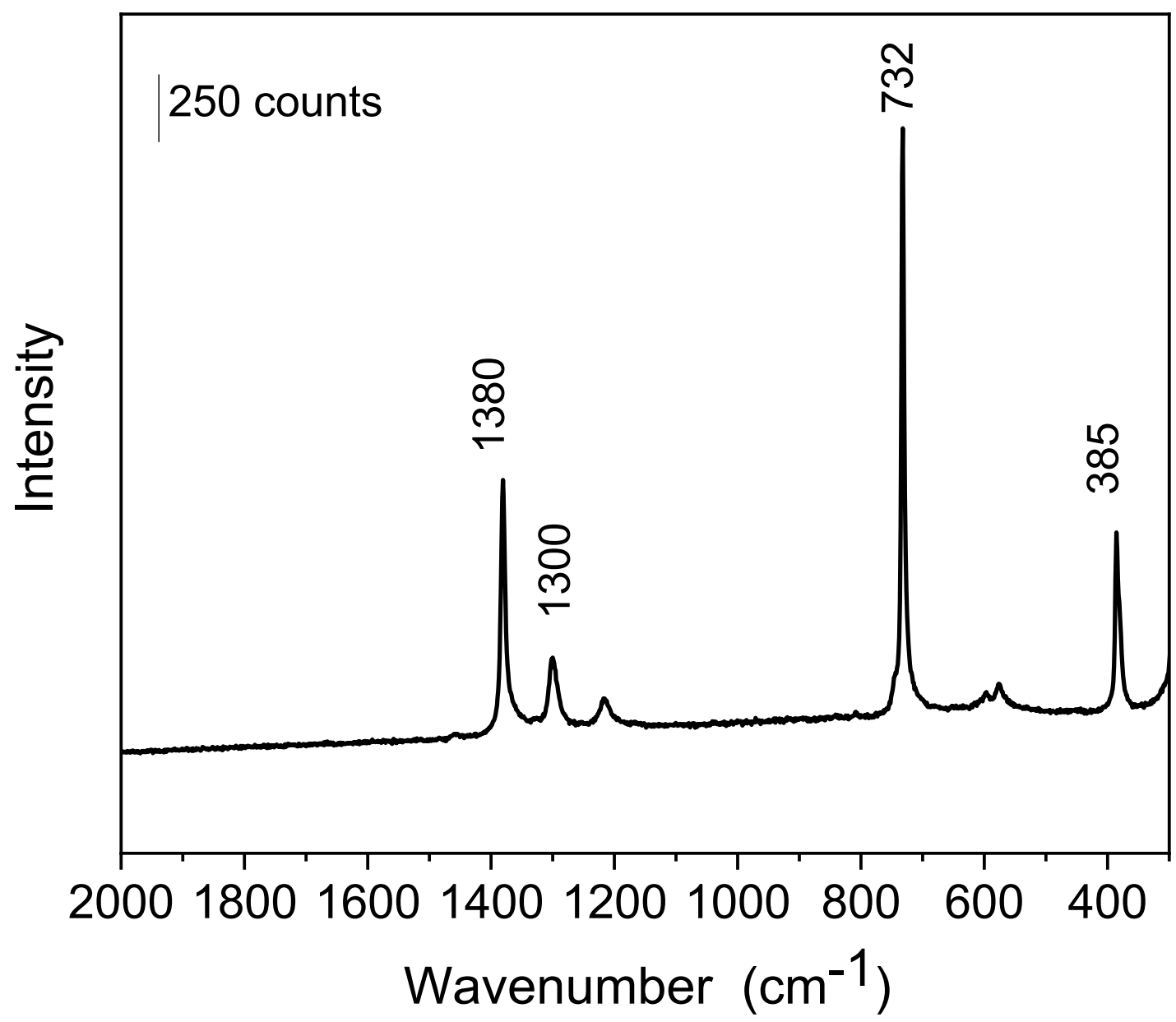

Figure S3: Raman spectrum of PTFE membrane.

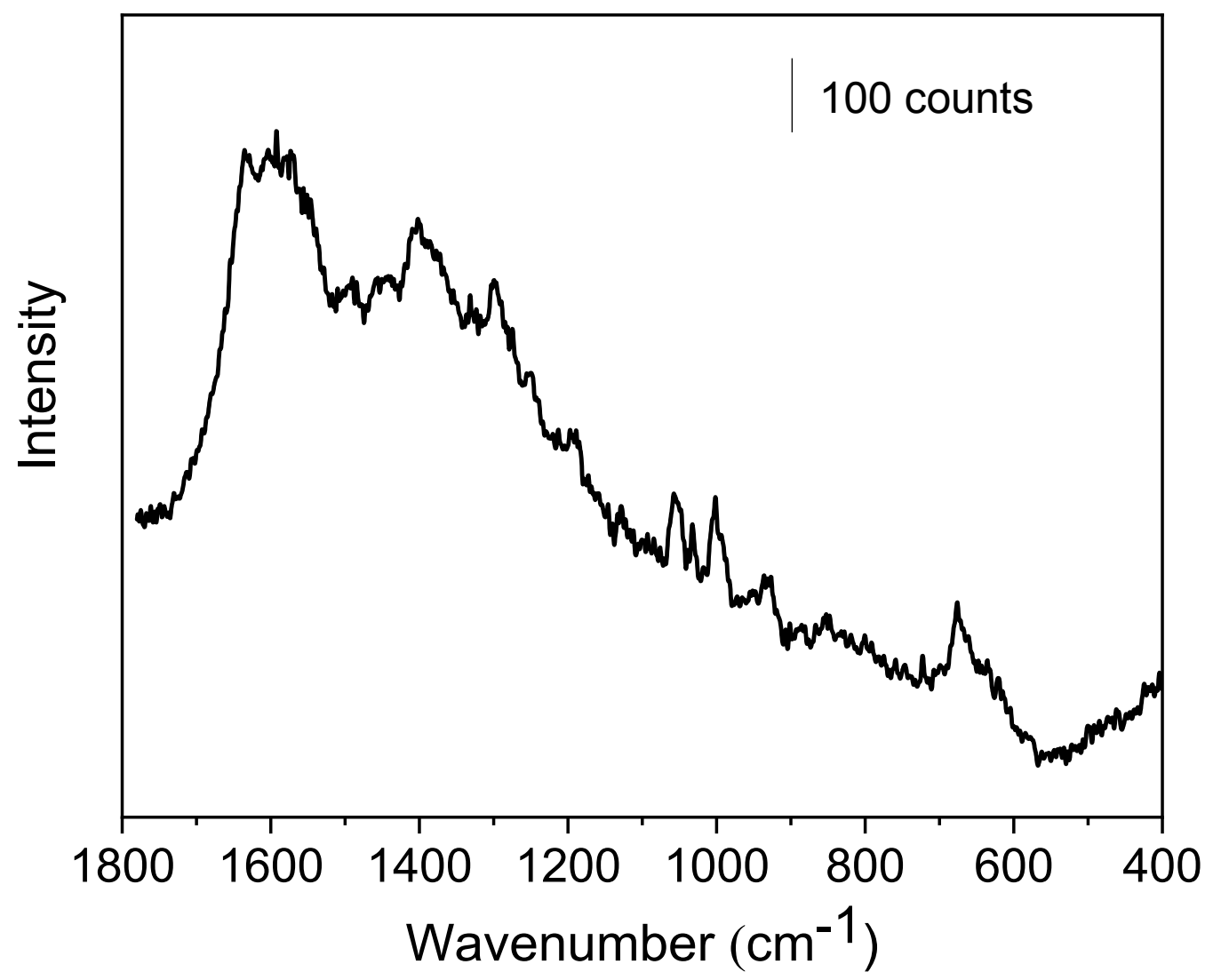

Figure S4: Raman spectrum of substate (PTFE-AgNP)

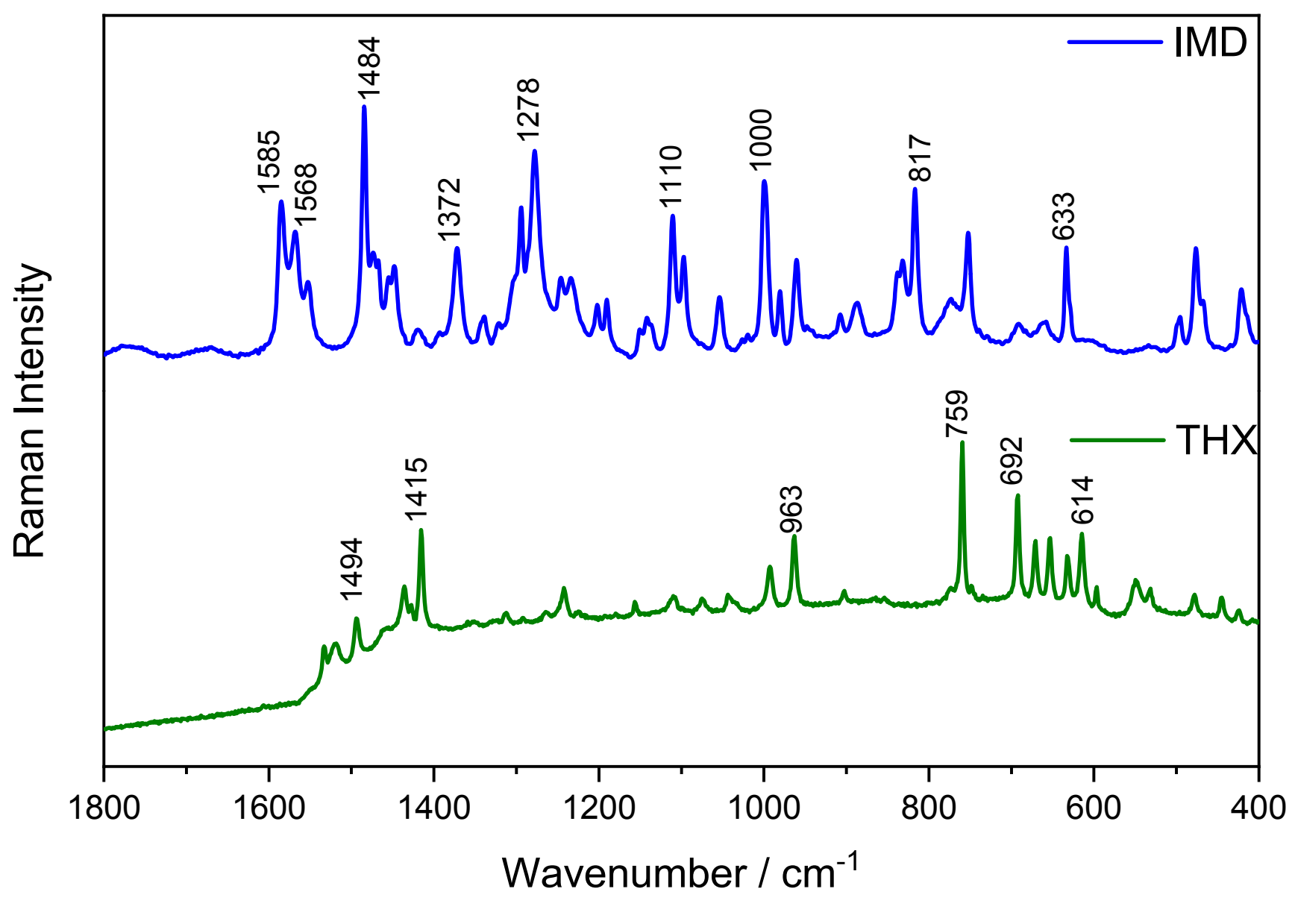

Figure S5: Raman spectra of imidacloprid and thiamethoxam in the solid state.

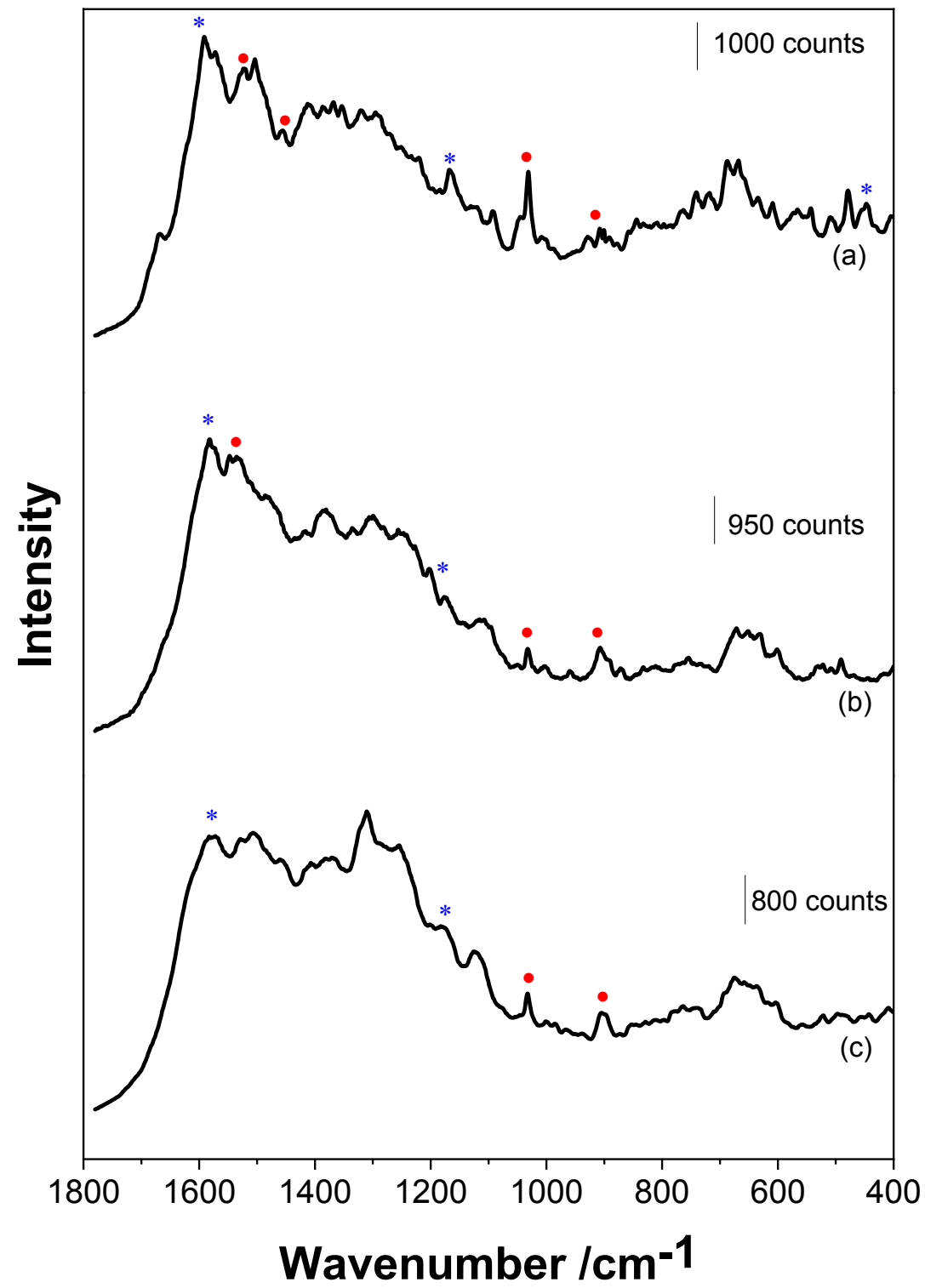

Figure S6: Mixture of pesticides on PTFE substrate at a concentration 1.0×10^{-4} mol L^{-1} (a) (1:1) (IMD: THX) (b) (1:3) (IMD: THX) (c) (3:1) (IMD: THX).

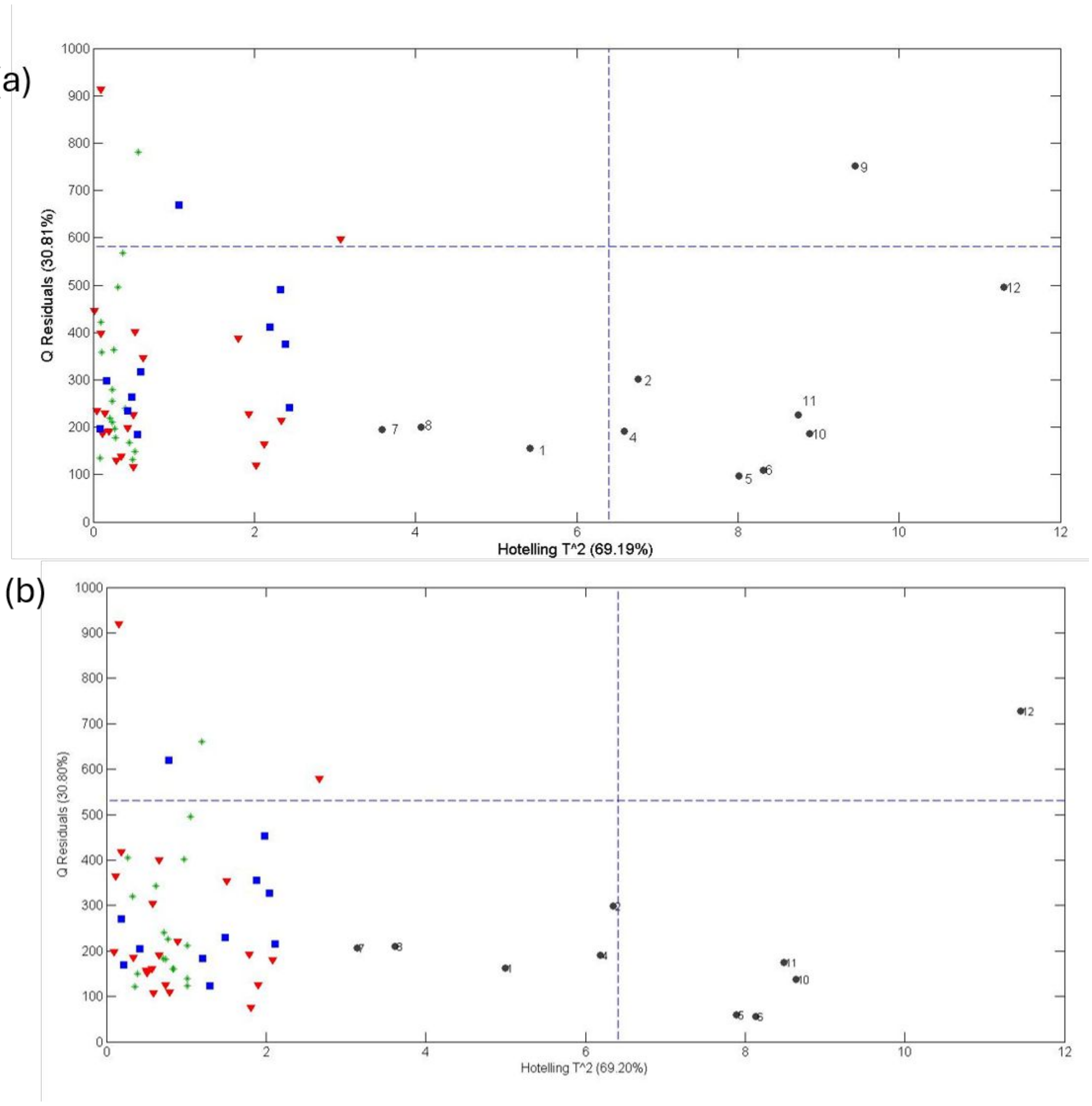

Figure S7: Q residual versus Hotelling's T² plots for the PCA model: (a) first round of outlier evaluation and (b) second round of outlier evaluation.

Table S1: Number of independent substrates analyzed for each pesticide concentration.

Pesticide	1.0×10^{-4} mol L^{-1}	1.0×10^{-5} mol L^{-1}	1.0×10^{-6} mol L^{-1}	1.0×10^{-7} mol L^{-1}	Total
IMD	9	4	3	6	22
THX	8	3	3	3	17
IMD + THX	3	3	3	3	12
Total	20	10	9	12	51
